# Inherited Dyslipidemic Splenomegaly: A Genetic Macrophage Storage Disorder Caused by Disruptive Apolipoprotein E (*APOE*) Variants

**DOI:** 10.3390/genes16030289

**Published:** 2025-02-27

**Authors:** Elise A. Ferreira, Machteld M. Oud, Saskia N. van der Crabben, Miranda Versloot, Susan M. I. Goorden, Clara D. M. van Karnebeek, Jeffrey Kroon, Mirjam Langeveld

**Affiliations:** 1 Department of Paediatrics, Emma Children’s Hospital, University of Amsterdam, 1105 AZ Amsterdam, The Netherlands; 2 United for Metabolic Diseases, 1105 AZ Amsterdam, The Netherlands; 3 Department of Human Genetics, Donders Institute for Brain, Cognition and Behaviour, Radboud University Medical Center, 6525 GA Nijmegen, The Netherlands; 4 Department of Human Genetics, Amsterdam University Medical Centres, Amsterdam Reproduction & Development, University of Amsterdam, 1105 AZ Amsterdam, The Netherlands; 5 Department of Experimental Vascular Medicine, Amsterdam Cardiovascular Sciences, Amsterdam UMC, University of Amsterdam, 1105 AZ Amsterdam, The Netherlands; 6 Center for Lysosomal and Metabolic Diseases, Department of Clinical Genetics, Erasmus University Medical Center, 3015 GD Rotterdam, The Netherlands; 7 Department of Clinical Chemistry, Erasmus Medical Center, 3015 GD Rotterdam, The Netherlands; 8 Amsterdam Cardiovascular Sciences, Atherosclerosis & Ischemic Syndromes, 1105 AZ Amsterdam, The Netherlands; 9 Laboratory of Angiogenesis and Vascular Metabolism, VIB-KU Leuven Center for Cancer Biology, VIB, 3000 Leuven, Belgium; 10 Laboratory of Angiogenesis and Vascular Metabolism, Department of Oncology, KU Leuven and Leuven Cancer Institute (LKI), 3000 Leuven, Belgium; 11 Department of Endocrinology and Metabolism, Amsterdam UMC, Research Institute Gastroenterology, Endocrinology & Metabolism (AGEM), University of Amsterdam, 1105 AZ Amsterdam, The Netherlands

**Keywords:** apolipoprotein E, splenomegaly, inherited lipemic splenomegaly, dysbetalipoproteinemia, N-palmitoyl-O-phosphocholine-serine (PPCS)

## Abstract

Background: Persistent splenomegaly, often an incidental finding, can originate from a number of inherited metabolic disorders (IMDs). Variants of *APOE* are primarily known as risk factors in terms of cardiovascular disease; however, severe dysfunction of APOE can result in a disease phenotype with considerable overlap with lysosomal storage disorders (LSDs), including splenomegaly and gross elevation of N-palmitoyl-O-phosphocholine-serine (PPCS). Methods: A case study (deep phenotyping, genetic and FACS analysis) and literature study was conducted. Results: The index patient, with a family history of early-onset cardiovascular disease, presented with splenic infarctions in a grossly enlarged spleen. The identified genetic cause was homozygosity for two *APOE* variants (c.604C>T, p.(Arg202Cys) and c.512G>A, p.(Gly171Asp); ε1/ε1), resulting in a macrophage storage phenotype resembling an LSD that was also present in the brother of the index patient. A FACS analysis of the circulating monocytes showed increased lipid content and the expression of activation markers (CD11b, CCR2, CD36). This activated state enhances lipoprotein intake, which eventually converts these monocytes/macrophages into foam cells, accumulating in tissues (e.g., spleen and vascular wall). A literature search identified seven individuals with splenomegaly caused by *APOE* variants (deletion of leucine at position 167). The combined data from all patients identified male gender, splenectomy and obesity as potential modifiers determining the severity of the phenotype (i.e., degree of triglyceride increase in plasma and/or spleen size). Symptoms are (partially) reversible by lipid-lowering medication and energy restricted diets and splenectomy is contra-indicated. Conclusions: Inherited dyslipidemic splenomegaly caused by disruptive *APOE* variants should be included in the differential diagnoses of unexplained splenomegaly with abnormal lipid profiles. A plasma lipid profile consistent with dysbetalipoproteinemia is a diagnostic biomarker for this IMD.

## 1. Introduction

Splenomegaly has a broad differential diagnosis, including infections, hematologic malignancies, hepatic disease, focal lesions in the spleen and autoimmune disease [1]. However, when the most common causes are ruled out and splenomegaly persists, the suspicion of an inherited metabolic disorder (IMD) grows. The most frequently diagnosed group of IMDs in adult patients presenting with unexplained persistent splenomegaly are the lysosomal storage diseases (LSDs) (see Appendix A Table A1 for a full overview of all IMDs associated with splenomegaly) [2]. LSDs are a heterogeneous group of disorders resulting from variants in genes encoding lysosomal degradative enzymes, associated activator proteins or lysosomal transporters that are essential for exporting degraded products from the lysosome [3]. Lysosomal enzyme malfunction leads to the buildup of specific substrates, eventually resulting in lysosomal dysfunction [2]. The specific type and pattern of affected organ systems points towards the most likely diagnosis (e.g., pulmonary involvement for Niemann–Pick type B (NPB; OMIM #607616) and corneal opacities for LCAT deficiency (OMIM #606967)) [2].

**Figure 1:**
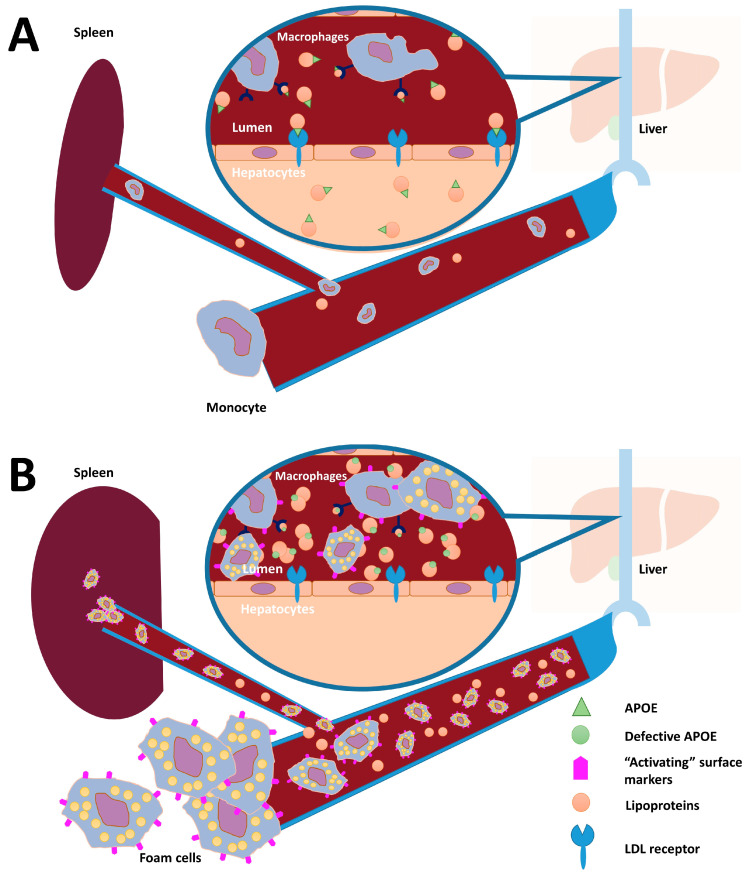
The theorized role of APOE in the prevention of atherosclerotic plaques, dyslipidemia and splenomegaly: (**A**) Under normal conditions, APOE is integrated into VLDL and chylomicrons during hepatic synthesis of these particles and facilitates the removal of “lipids” (converted VLDL components, i.e., LDL) from plasma by functioning as a ligand for LDL receptors (LDLRs) on hepatocytes. Macrophages secrete APOE and simultaneously absorb APOE-containing lipoproteins. (**B**) In patients with *APOE*-compromising variants, APOE has decreased affinity for LDL receptors. An elevated amount of lipoproteins in plasma (total cholesterol and triglycerides) result from the impaired clearance of chylomicron and VLDL remnants by hepatocytes. The presence of large quantities of lipids will lead to the increased expression of macrophage-activating surface markers (a.o. CD11b, CCR2, CD36) and stimulate them to actively load lipids (the uptake of lipid particles is depicted by the yellow particles in foam cells), eventually converting into foam cells. Finally, these foam cells accumulate in the bloodstream and various tissues, including the spleen, eventually resulting in splenomegaly.

A subset of IMDs that can present with splenomegaly in adulthood are also characterized by marked changes in the lipoprotein profile (e.g., Tangier disease (OMIM #205400) and cholesterol ester storage disease (OMIM #278000)). The combination of unexplained dyslipidemia and splenomegaly is therefore a strong indicator for an underlying genetic cause [1]. A combination of more general biomarkers (e.g., chitotriosidase activity, a macrophage activation marker which is elevated in, among other diseases, Gaucher (OMIM #230800), Niemann–Pick type B and C disease (NPC; OMIM #257220)) and highly specific biomarkers (e.g., glucosylsphingosine for Gaucher disease) can contribute to identifying the correct diagnosis. The diagnosis can subsequently be confirmed by the measurement of the activity of specific enzymes and genetic testing. In the group of “splenomegaly-causing LSD”, Niemann–Pick disease type C takes a special place since it is not a specific enzyme deficiency but a cholesterol trafficking disorder. It shares part of its clinical presentation with other LSDs: splenomegaly, elevated macrophage activation markers and, in some cases, the presence of foamy macrophages in bone marrow. For Niemann–Pick disease type B and C, N-palmitoyl-O-phosphocholine-serine (PPCS, initially named lysosphingomyelin-509) is a sensitive biomarker, especially when grossly elevated. To subsequently discriminate between these two types of Niemann–Pick disease, the lyso-sphingomyelin value can be determined, which is markedly increased in patients with Niemann–Pick disease type B [4]. PPCS is detectable in various matrices (cerebrospinal fluid, blood, and liver products) and belongs to a class of newly characterized lipids. Its biosynthetic pathway, however, has yet to be uncovered [5]. Formerly thought to be NPC specific, this marker has recently also been shown to be elevated in several other disorders, including a congenital disorder of glycosylation, ATP6AP1-CDG (OMIM #300197) [5]. In this article, we show that the differential diagnosis for an adult presenting with a macrophage storage disorder phenotype, including elevated levels of PPCS, should be broadened with dyslipidemic splenomegaly, caused by pathogenic variants in *APOE* (Figure 1). We base this conclusion on the genetic and biochemical findings in our index patient, a 37-year-old man with splenomegaly, his brother and functional studies performed with the macrophages of the index patient. In addition, we performed a meta-analysis of comparable cases reported in literature to find additional evidence of the relationship between pathogenic *APOE* variants and the inherited dyslipidemic splenomegaly phenotype. 

## 2. Materials and Methods

### 2.1. Subjects

#### 2.1.1. Brief Description of the ZOEMBA Study

The ZOEMBA (Dutch full name: ZOektocht naar Erfelijke MetaBole Aandoeningen) is a prospective, diagnostic, multicentre cohort study. Genomics (whole-exome sequencing (WES) reanalysis/whole-genome sequencing (WGS)) and untargeted metabolomic technologies (next-generation metabolomics screening in both plasma and blood spots [6,7] are combined with extensive phenotyping to find the genetic cause in patients with an unexplained phenotype suggestive of an IMD in whom standard of care diagnostics did not yield a diagnosis.

#### 2.1.2. Ethics Approval

The ZOEMBA study protocol and informed consent form was reviewed and approved by the medical ethics committee of the Amsterdam UMC (NL67721.018.19) and registered at https://clinicaltrials.gov (accessed on 16 January 2025) (NCT06200142). The study complied with the Declaration of Helsinki, Good Clinical Practice, and the regulatory requirements in the Netherlands. The authors have obtained written consent forms from both patients (index patient and his brother).

#### 2.1.3. Beacon Protocol

All three control subjects (matched to the index patient for both age and gender) were recruited via the “*Beacon protocol*”. This collaboration initiative of the biomedical departments in the Amsterdam UMC, reviewed and approved by the Amsterdam UMC medical ethics committee, recruits healthy volunteers for bodily fluid donations. All control subjects signed informed consent via the *Beacon protocol* prior to inclusion. 

#### 2.1.4. Genomic Analysis

WES was performed for the index patient on an Illumina HiSeq4000 sequencer (Illumina, San Diego, CA, USA). Reads were mapped along the GRCh37 (HG19) reference genome using BWA v0.7.12 and duplicate marked using Picard v1.90. Subsequently, variant calling of single-nucleotide variants (SNVs) and small indels was carried out using GATK v.3.4-46. Short-tandem repeats (STRs) were analyzed using Expansion Hunter v3.1.2. with default settings. Variant annotation was performed using a custom diagnostic annotation pipeline. Low-quality variants and variants with a frequency >1% in dbSNP, GnomAD v2.1 or the in-house database were filtered out. The data were first analyzed in a diagnostic setting using a virtual customized gene panel including *SMPD2*, *SMPD3*, *SMPD4*, *CTSB* and *CTSL.* Six months later, reanalysis of the WES data was performed as part of the ZOEMBA study, where an open exome analysis was performed. In the reanalysis, all nonsense variants and missense variants with a CADD score > 20, phyloP > 2.7 or spliceAI ≠ 0 were prioritized. Subsequently, for known disease genes, the genotype–phenotype correlation was studied. 

#### 2.1.5. Nomenclature of APOE Isoforms

*APOE* (NM_001302688.2) is known to have two polymorphisms, rs429358 and rs7412, resulting in three common protein isoforms (APOE-ε2, APOE-ε3 or APOE-ε4). The APOE-ε3 (Cys156/Arg202, formerly annotated as Cys112/Arg158) is considered the wild-type form and accounts for approximately ~60–75% of all alleles [1]. The other APOE isoforms have less affinity for lipoprotein receptors on cells, thus are perceived as less functional or even defective [1,8,9]. APOE-ε2 (Cys156/Cys202, formerly annotated as Cys112/Cys158) constitutes ~10% of all alleles, and functional tests have demonstrated that APOE-ε2 binds with <2% affinity to the LDL-receptor compared to APOE-ε3 [10,11,12]. However, despite ~1% of the population being homozygous for APOE-ε2, only ~5% of these homozygotes develop dysbetalipoproteinemia, mostly in the presence of other risk factors for dyslipidemia [13]. APOE-ε4 (Arg156/Arg202, formerly annotated as Arg112/Arg158), accounting for approximately 15% of all alleles, has been associated with an increased risk for Alzheimer’s disease and will not be discussed in this paper [12,14]. 

On top of these relatively common isoforms, additional rare (pathogenic) variants in *APOE* can present, with a possible additive negative effect on the functionality of the APOE protein. A rare occurrence is the presence of a second variant in the *APOE-ε2* variant, causing a second amino acid change (glycine to asparagine at position 171, formerly annotated as 127) in APOE-ε2 isoform (Cys156/Cys202), thus yielding a protein with two amino acid changes compared to wild-type APOE-ε3, which is referred to as the APOE-ε1 isoform [9,15]. Similarly to APOE-ε2, APOE-ε1 has a reduced binding ability (<4%) to the LDL-receptor compared to APOE-ε3 [9]. The *APOE-ε1* allele is also known as the “*Weisgraber allele*”. Its presence (in the heterozygous state) has been associated with dysbetalipoproteinemia [9,15,16,17]. 

### 2.2. Functional Assays in Monocytes of the Index Patient and Three Healthy Control Subjects

#### 2.2.1. Nomenclature of Monocyte Fractions 

Human monocytes can be subdivided into three fractions, each having different functions in the immune response, based on their expression of surface markers: *classical*, *intermediate* and *non-classical* monocytes [18]. CD14^++^CD16 monocytes are classified as *classical*, CD14^++^CD16^+^ are *intermediate* and CD14^+^CD16^+^ are classed as *non-classical*. *Classical* monocytes are involved in phagocytosis, adhesion, migration and anti-microbial responses. *Intermediate* monocytes regulate apoptosis, transendothelial migration and antigen presentation. Lastly, the function of *non-classical* monocytes consists of complement and FcR-mediated phagocytosis, trans-endothelial migration, adhesion and anti-viral responses [18]. 

#### 2.2.2. Intracellular Lipid Droplet Accumulation

Nile Red (9-diethylamino-5H-benzo [α]phenoxazine-5-one) was dissolved in DMSO (318 µg/mL => 1 mM) and filtered through a 0.22 µM syringe to reduce background and create a homogenous solution. The stock solution was diluted in PBS to a final concentration of 10 µM (protect from light). Microscope glass was coated with fibronectin after drawing a circle (diameter 1.5 cm) with a DAKO pen for at least 1 h. Cells were added 100–200 µL (0.5 × 10^5^ cells in total) and incubated for 1 h at 37 °C, 5% CO_2_. Cells were then fixed for 15 min at room temperature (4% formaldehyde). Cells were washed with PBS and stored at 4 °C. Cells were permeabilized for 5 min with 0.1% Triton X-100, once washed with PBS and incubated with 3.3 µM Nile Red for 15 min. Cells were washed with PBS and mounted (DAKO fluorescent mounting media). Monocytes were imaged with the Leica TCS SP8 Confocal microscope (Leica Microsystems GmbH, Mannheim, Germany) (63× Oil objective was used (phospholipids were excited at 590 (600–700 nm) and neutral lipids at 488 (500–580 nm). Quantification was performed by counting the total number of monocytes with lipid droplets per field of view (FOV), as well as the number of lipid droplets per positive monocyte in 6–10 FOVs. To visualize all lipid droplets, z-stack images of 1 µM per stack were made (1024 pixels × 1024 pixels).

#### 2.2.3. Flow Cytometry 

Whole blood was collected from the index patient and age- and gender-matched healthy control subjects. Red blood cells were lysed using red blood cell lysis buffer 10× (eBioscience, San Diego, CA, USA) (followed by staining of white blood cells for the surface markers CCR2, CD11c, CD36, CD29, CD18, CX3CR1, TLR2, CD11b, SR-A, TLR4, HLA-DR, CD14, CD16 and IVIG (See Appendix B Table A2|Antigen overview). Fluorescent intensity was measured using a FACS CANTO II (BD) and analyzed with FlowJo software version 10.6. Subsequently, the monocyte area was gated based on forward and side scatter, HLA-DR and CD14^+^ and/or CD16^+^. Then, monocytes were classified as *classical* (CD14^++^CD16^−^), *intermediate* (CD14^++^CD16^+^) or *non-classical* (CD14^+^CD16^+^). The expression of cell surface markers was calculated as the delta geometric mean (∆GM). ∆GM = GM surface staining—GM unstained control. 

### 2.3. Literature Search 

**Figure 2:**
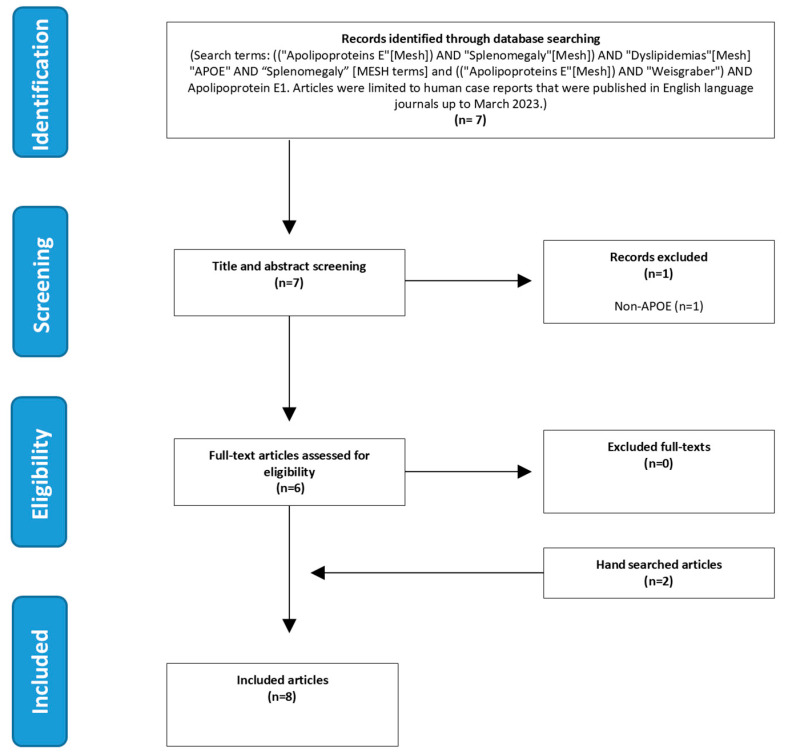
PRISMA Flowchart meta-analysis.

A retrospective case-report-based meta-analysis was performed, looking for case descriptions of patients with splenomegaly and variants in *APOE*. In addition, we searched for reports on individuals with heterozygous or homozygous *Weisgraber allele(s).* The meta-analysis was performed according to the “Preferred Reporting Items for Systematic Reviews and Meta-Analyses statements” [19,20]. We searched for eligible articles using two combinations of Medical Subject Headings (MeSH) and free text from the inception of these databases to 29 March 2023. We also hand-searched references of all included studies to identify review articles and related meta-analyses. The search was performed with the following terms: ((“Apolipoproteins E”[Mesh]) AND “Splenomegaly”[Mesh]) AND “Dyslipidemias”[Mesh] “APOE” AND “Splenomegaly”[MESH terms] and (((“Apolipoproteins E”[Mesh]) AND “Weisgraber”) AND Apolipoprotein E1). E.A.F. conducted the literature search and E.A.F. and M.L reviewed the titles, abstract, and full-text articles to determine if they met the inclusion criteria (*APOE* variants AND splenomegaly OR Weisgraber allele/APOE-ε1 WITH/WITHOUT splenomegaly). Ambiguities were resolved through discussion with co-author M.L. Only publications written in English were included (See Figure 2, PRISMA flowchart).

### 2.4. Statistical Analysis 

Boxplots and statistical comparisons were constructed using IBM SPSS statistics, version 26. Statistical comparisons between the non-parametric groups (literature meta-analysis) were performed by a Wilcoxon Signed Rank test. A two-sided *p*-value of ≤ 0.05 was considered significant. 

## 3. Results

### 3.1. Case Report

A 37-year-old male (index patient, Table 1 and Table 2) of Moroccan descent and from consanguineous parents presented with acute sensations of pain in the upper left quadrant of the abdomen due to splenic infarction in a grossly enlarged spleen. Prior medical history included hypersplenism since early childhood (at this time no cause was established and monitoring was discontinued in his teens) and obesity (BMI 33 kg/m^2^). The family history was positive for coronary artery disease (CAD). Six out of 14 siblings of the patient’s father suffered from CAD from a young age, including the father himself, who had died prematurely from acute pancreatitis of an unknown etiology (triglyceride levels were not determined at the time of his hospital admission). Common causes of splenomegaly, such as viral infections, were excluded, and a bone marrow biopsy was performed because of suspicion of a hematological malignancy. The histological examination showed macrophage foam cells, suggestive of a LSD. The lysosphingolipid profile in plasma showed a pronounced elevation of N-palmitoyl-O-phosphocholine-serine (PPCS; 547 nmol/L, reference range 1.3–32.2 nmol/L) and a marginally elevated lyso-sphingomyelin (7.4 nmol/L, reference range 0.2–3.0 nmol/L). Furthermore, chitotriosidase activity was elevated (901 nmol/h/mL, reference range 0–90 nmol/h/mL), as were oxysterol levels: 7-ketocholesterol (0.75 umol/L, reference range 0–0.52) and cholestane-3-β,5-alfa,6-β-triol (0.29 µmol/L, reference range 0–0.058). Sphingomyelinase activity was slightly decreased (9 nmol/h/mg, reference range 10–53 nmol/h/mg). Filipin staining in fibroblasts was negative. 

The clinical phenotype (enlarged spleen, foamy macrophages in bone marrow) and the metabolite pattern (massively elevated PPCS, elevated chitotriosidase activity and increased oxysterols) resembled that of Niemann–Pick type C; however, no neurological abnormalities (specifically no supranuclear gaze palsy) were found with detailed clinical examination. Additionally, no pathogenic variants were detected in *NPC1*/*NPC2* or *SMPD1* by Sanger sequencing and Multiplex Ligation-dependent Probe Amplification (MLPA). Due to the high suspicion of sphingolipid metabolism pathology in the index patient, exome analysis with a virtual customized gene panel was performed (*SMPD2*, *SMPD3*, *SMPD4*, *CTSB* and *CTSL*), which did not identify any (potentially) pathogenic variants. Next, an open WES reanalysis was performed as part of the ZOEMBA study. A shortlist of 17 variants was generated with rare (<1% MAF) nonsense variants or missense variants with a CADD score > 20 (see Appendix B Table A3 for a full overview of detected, potentially pathogenic, variants). For known disease genes, the phenotype–genotype correlation was checked, which led to the prioritization of a homozygous missense variant in *APOE*.

**Figure A1:**
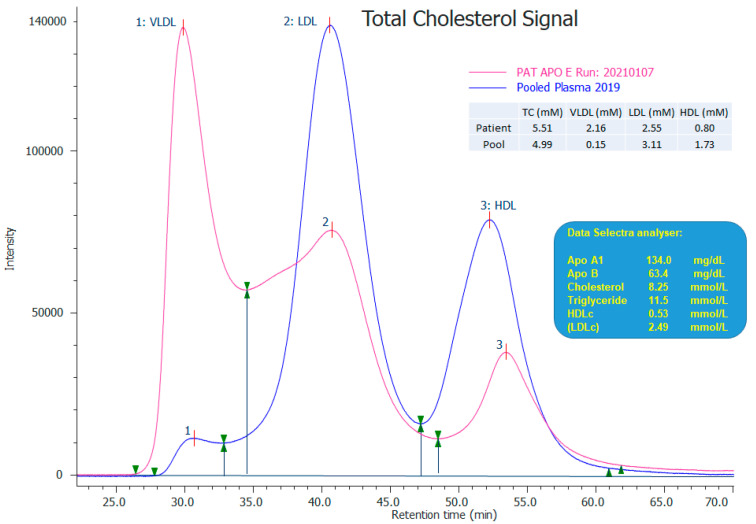
Plasma lipid profile of index patient.

The detected homozygous missense variant c.512G>A p.(Gly171Asp), classified as class 3 (a *variant of unknown significance*) in ClinVar, was prioritized due to the previously reported association with splenomegaly and foam cells in bone marrow in patients with other *APOE* variants in literature [8,21]. In addition, the index patient was shown to be homozygous for *APOE-ε2* rs7412 c.604C>T p.(Arg202Cys). This second variant was initially not prioritized in the analysis because of the relative high frequency in the healthy population (GnomAD v.2.1.1, MAF 12% heterozygous and 0.5% homozygous); however, since this patient was now homozygous for both variants, and therefore homozygous for the *Weisgraber allele* (*APOE-ε1*), all *APOE* variants were considered interesting for the phenotype (see discussion). Additionally, a variant in *NPC1* (NM_000271.5) c.463+19A>G was identified, but this variant has been reported as benign in ClinVar and thus deemed not causative of the phenotype. Due to the identification of the homozygous Weisgraber alleles, a lipid panel was acquired according to the method described by Heidemann et al. [22]; this can only be performed by specialized laboratories using ultracentrifugation. The lipid panel showed (after removal of chylomicrons and compared to pooled plasma of control subjects) a total cholesterol (TC) of 5.5 mmol/L (pool 4.99); very-low-density lipoprotein (VLDL) 2.16 mmol/L (pool 0.15); low-density lipoprotein (LDL) 2.55 mmol/L (pool 3.11); high-density lipoprotein (HDL) 0.80 mmol/L (pool 1.73) (SeeAppendix B Figure A1|Plasma lipid profile). Next, we offered to include both brothers of the index patient in the ZOEMBA study as they exhibited similar phenotypes, to which the youngest brother agreed; his details are discussed below. 

The brother (Table 1 and Table 2), a 26-year-old male, had no relevant medical history except for obesity (BMI 30 kg/m^2^). An ultrasound of the spleen revealed a slightly enlarged spleen (12.4 cm, upper limit of normal). Laboratory studies showed significant elevations in total cholesterol (9.0 mmol/L, reference range < 3.4 mmol/L); total triglycerides (3.2 mmol/L, reference range < 2.0 mmol/L); a normal HDL cholesterol level (1.2 mmol/L, reference range > 0.9 mmol/L); elevated PPCS (81.3 nmol/L, reference range 1.3–32.2 nmol/L) and lyso-sphingomyelin (4.9 nmol/L, reference range 0.2–3.0 nmol/L). Targeted Sanger sequencing of the two homozygous *APOE* variants and the heterozygous *NPC1* variant detected in the index patient were also identified in his brother. 

After diagnosis, lipid-lowering medication (Atorvastatin and Ciprofibrate) was started in both brothers. Additionally, an energy-restricted and saturated-fat-restricted diet and lifestyle changes (i.e., regular exercise and cessation of smoking) were advised. The patients were contra-indicated for splenectomy. Additionally, the whole family was offered genetic counseling.

**Table 1:** Characteristics of patients with splenomegaly caused by variants in the *APOE* gene (NM_001302688.2).

Patient ID (Reference)	Case Type	Age (Years), Sex	*APOE* Allele 1 (Isoform) *	*APOE* Allele 2 (Isoform) *	BMI > 25 kg/m^2^	Cardiovascular Involvement	Splenomegaly	Plasma Lipid Spectrum (mmol/L) #		Foam Cells (Location of Infiltration)
								TG	TC	
**Index (current study)**	**Index patient**	**37, M**	**p.(Gly171Asp)/p.(Arg202Cys) (ε1)**	**p.(Gly171Asp)/p.(Arg202Cys) (ε1)**	**Y**	**N**	**Y**	11.7	7.2	Y (Bone marrow and spleen)
**Brother (current study)**	Brother of index patient	26, M	p.(Gly171Asp)/p.(Arg202Cys) (ε1)	p.(Gly171Asp)/p.(Arg202Cys) (ε1)	Y	N	Y	3.2	9.2	NM
**A [1]**	Proband	76, M	p.(Leu167del) (ε3)	p.(Arg202Cys) (ε2)	Y	Severe ischemic heart disease; essential hypertension; systolic ejection murmer (grade 2/6)	Y	16.2	6.2	N
**B [12]**	Proband	49, M	p.(Leu167del) (ε3)	p.(Arg202Cys) (ε2)	Y	N	Y	<2.0	Normal	Y (Spleen)
**C [21]**	Proband	47, M	p.(Leu167del) (ε3)	p.(Arg202Cys) (ε2)	Y	N	Y	13.6	4.8	Y (Bone marrow and spleen)
**D [21]**	Brother of C	NR, M	p.(Leu167del) (ε3)	WT (ε3)	NR	Ischemic heart disease	Y	1.9	5.7	NR
**E [8]**	Proband	29, M	p.(Leu167del) (ε3)	WT (ε3)	Y	Ischemic heart disease	Y	2.1	2.3	Y (Spleen)
**F [8]**	Mother of E	NR, F	p.(Leu167del) (ε3)	p.(Arg202Cys) (ε2)	NR	Ischemic heart disease	Y	1.7	4.6	NR
**G [8]**	Proband	49, M	p.(Leu167del) (ε3)	WT (ε3)	Y	Ischemic heart disease	Y	4.3	3.8	Y (Spleen)

* Re-annotated to current standards. ^#^ Untreated lipid spectrum (and prior to splenectomy, if relevant), apart from patient C, who was treated with lipid-lowering therapy). TG, triglyceride (mmol/L); TC, total cholesterol (mmol/L); NM, not measured; NR, not reported; N, no; Y, yes; M, male; F, female; WT, wild-type variant (APOE-ε3). The patients in this current study are depicted by a grey background in the table.

**Table 2:** Characteristics of patients with one or two APOE-ε1 isoforms (NM_001302688.2).

Patient ID (Reference)	Case Type	Age (Years), Sex	*APOE* Allele 1 (Isoform) *	*APOE* Allele 2 (Isoform) *	BMI > 25 kg/m^2^	Cardiovascular Involvement	Splenomegaly	Plasma Lipid Spectrum (mmol/L ^#^		Foam Cells (Location of Infiltration)
								TG	TC	
**Index** **(current study)**	**Index patient**	**37, M**	**p.(Gly171Asp)/p.(Arg202Cys)** **(** **ε** **1)**	**p.(Gly171Asp)/p.(Arg202Cys)** **(** **ε** **1)**	**Y**	**N**	**Y**	11.7	7.2	Y (Bone marrow and spleen)
**Brother** **(current study)**	Brother of index patient	26, M	**p.(Gly171Asp)/p.(Arg202Cys)** **(** **ε** **1)**	**p.(Gly171Asp)/p.(Arg202Cys)** **(** **ε** **1)**	Y	N	Y	3.2	9.2	NM
**H [9]**	Proband	49, M	WT (ε3)	p.(Gly171Asp)/p.(Arg202Cys) (ε1)	Y	Inverted T-waves on ECG	NR	12.0	5.3	NR
**I [16]**	**Proband**	**31, M**	**p.(Gly171Asp)/p.(Arg202Cys)** **(** **ε** **1)**	**p.(Gly171Asp)/p.(Arg202Cys)** **(** **ε** **1)**	**N**	**N**	**NR**	**4.6**	**19.5**	**NR**
**J [16]**	**Sister of I**	**16, F**	**p.(Gly171Asp)/p.(Arg202Cys)** **(** **ε** **1)**	**p.(Gly171Asp)/p.(Arg202Cys)** **(** **ε** **1)**	**N**	**N**	**NR**	**1.8**	**4.2**	**NR**
**K [17]**	Proband	42, F	p.(Gly171Asp)/p.(Arg202Cys) (ε1)	p.(Arg202Cys) (ε2)	Y	N	NR	5.7	9.4	NR
**L [17]**	Son of K	15, M	p.(Gly171Asp)/p.(Arg202Cys) (ε1)	p.(Arg202Cys) (ε2)	Absence significant obesity reported	NR	NR	4.2	7.7	NR
**M [17]**	Son of K	13, M	p.(Gly171Asp)/p.(Arg202Cys) (ε1)	WT (ε3)	NR	NR	NR	0.5	4.8	NR
**N [17]**	Son of K	9, M	p.(Gly171Asp)/p.(Arg202Cys) (ε1)	p.(Arg202Cys) (ε2)	NR	NR	NR	1.6	4.5	NR
**O [17]**	Uncle of K	78, M	p.(Gly171Asp)/p.(Arg202Cys) (ε1)	p.(Arg202Cys) (ε2)	NR	N	NR	2.9	9.6	NR
**P [15]**	**Proband**	**60, F**	**p.(Gly171Asp)/p.(Arg202Cys)** **(** **ε** **1)**	**p.(Gly171Asp)/p.(Arg202Cys)** **(** **ε** **1)**	**Y**	**Hypertension (up to 240/150 mmHg)**	**NR**	**2.9**	**8.2**	**NR**

* Re-annotated to current standards. ^#^ Untreated lipid spectrum (apart from patients H and I, who were treated with lipid-lowering therapy and patient K who was on a lipid-lowering diet). TG, triglyceride (mmol/L); TC, total cholesterol (mmol/L); NM, not measured; NR, not reported; N, no; Y, yes; M, male; F, female; WT, wild-type variant (APOE-ε3). Homozygous APOE-ε1 carriers are in bold print. The patients in this current study are depicted by a grey background in the table.

### 3.2. Functional Studies in Monocytes of Index Patient

#### 3.2.1. Lipid Droplet Count

**Figure 3:**
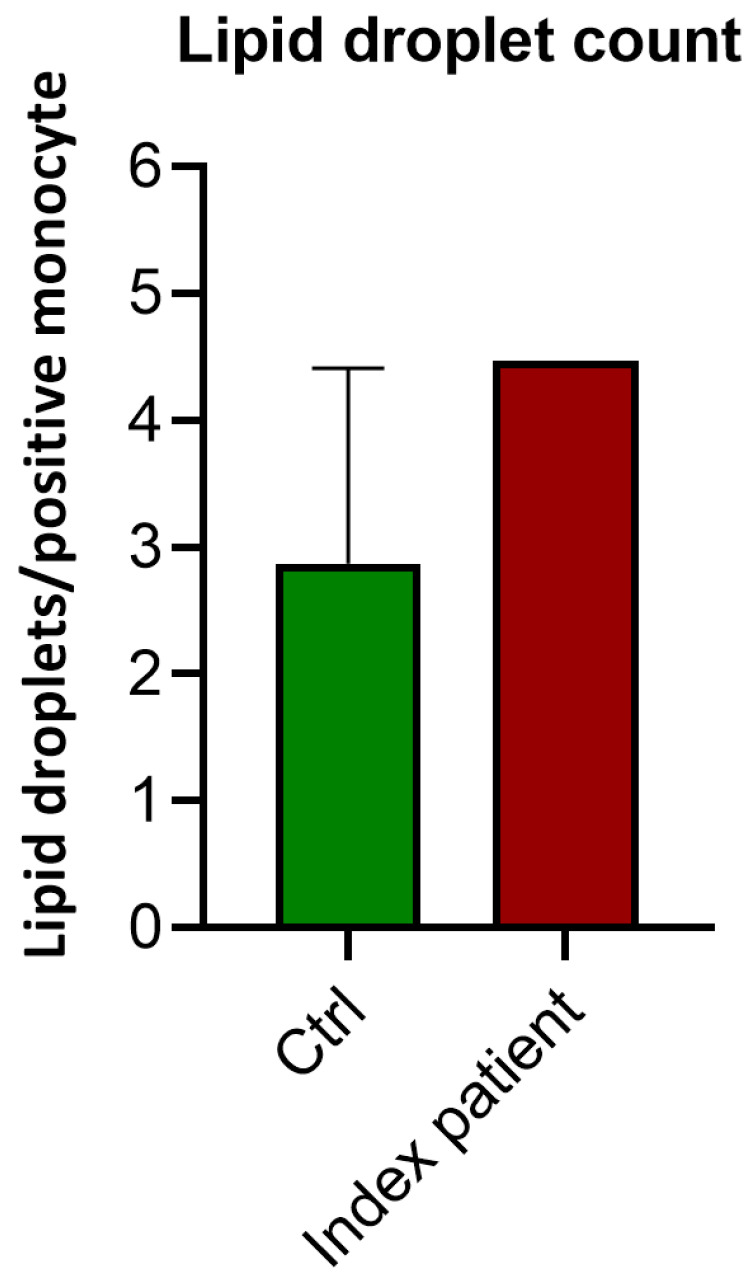
Lipid droplet count in the monocytes of the index patient and three control subjects: Median and range depicted.

In order to confirm that the disruption in APOE function leads to lipid accumulation in monocytes, we compared the monocytes of the index patient to those of age- and gender-matched control subjects. The lipid droplet per monocyte count was higher in the index patient (4.47 lipid droplets/monocyte) compared to the three healthy control subjects (median of 2.87 lipid droplets/monocyte (range 2.29–4.41) (Figure 3). 

#### 3.2.2. Flow Cytometry Analysis of Monocytes

**Figure 4:**
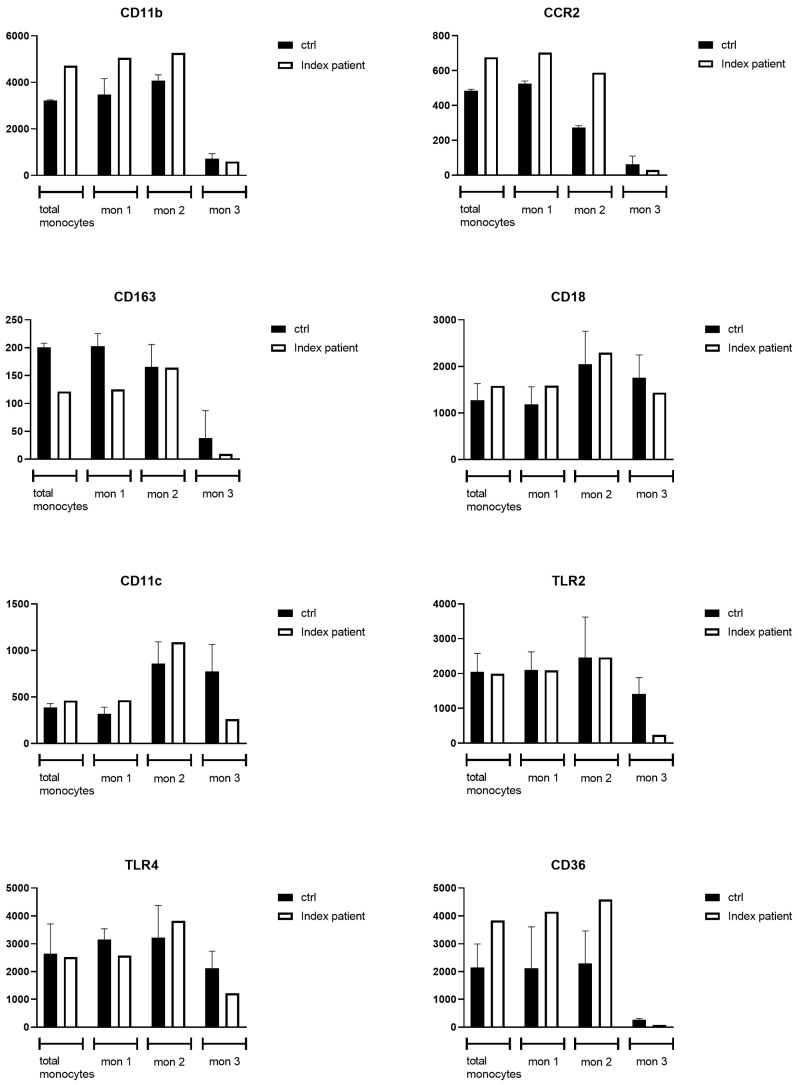
FACS analysis of monocyte surface marker expression: ctrl, pooled healthy control subjects (n = 3) and the index patient (n = 1); Mon, monocyte (mon 1, *classical* pro-inflammatory monocytes; mon 2, *intermediate* monocytes and mon 3; *non-classical* monocytes); CD, cluster of differentiation; CCR, chemokine receptor; TLR, toll-like receptor. Median and range depicted.

To study the effect of the lipid accumulation in the monocytes of the index patient, the activation state was analyzed by measuring the expression of monocyte surface markers. Flow cytometry (FACS) results (Figure 4) (see Appendix C for a full overview of all FACS data) showed an increased expression of CD11b, CCR2 and CD36 on the monocytes of the index patient compared to those of healthy control subjects (n = 3), especially in the classical and intermediate monocyte fraction. The CD11b, CCR2 and CD36 expression levels were 5059 ∆GM, 703 ∆GM, 4151 ∆GM in the index patient on the mon 1/classical monocyte fraction, compared to 3620 ∆GM (range 2746–4086), 527 ∆GM (range 511–540) and 2118 ∆GM (range 1655–3610) for the healthy control subjects. CD11b, CCR2 and CD36 showed an expression of 5262 ∆GM, 588 ∆GM, 4594 ∆GM in the index patient on mon 2/intermediate monocyte fraction, compared to an expression of 4006 ∆GM (range 3845–4345), 274 ∆GM (range 204–283) and 1636 ∆GM (range 1612–3644) for the healthy control subjects, respectively. The expression of the anti-inflammatory marker CD163, was reduced in patient monocytes, especially in classical (125 ∆GM) and non-classical (9 ∆GM) monocyte fractions compared to the expression in the classical (207 ∆GM; range 178–223) and mon 3/non-classical (23 ∆GM; range −2.0–93) monocyte fractions of the healthy control subjects [23]. The expression of CD11c and the expression of the pattern recognition receptors, TLR2 and TLR4, were reduced in the patient’s non-classical monocyte fractions only, 262 ∆GM, 241 ∆GM, 1213 ∆GM, respectively, compared to the median expression in the non-classical monocytes of the healthy control subjects of 706 (range 523–1094), 1152 (range 1140–1954), 2427 (range 1399–2519) ∆GM, respectively. 

### 3.3. Meta-Analysis of Literature

To find additional evidence for the relationship between pathogenic *APOE* variants and the inherited dyslipidemic splenomegaly phenotype present in our index patient, a meta-analysis of cases reported in the literature was conducted. As shown in Figure 2, a total of seven articles were identified in the initial search. One article was excluded after title and abstract screening because it reported on a different gene. After a full-text assessment of the remaining six articles, all fulfilled the inclusion criteria and were included in this meta-analysis. Two more articles were included by hand-searching the references of the initial set of included articles. The eight analyzed articles reported on a total of seven patients with splenomegaly caused by the *APOE* variant (s). Nine of the described patients presented with *APOE-ε1*; three patients were homozygous and six were heterozygous. 

**Figure 5:**
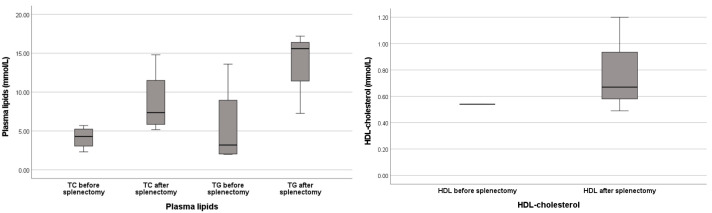
Lipid profile before and after splenectomy in cases with inherited dyslipidemic splenomegaly reported in the literature: TG, triglycerides (mmol/L); TC, total cholesterol (mmol/L); HDL, high-density lipoprotein (mmol/L). Lipid profiles shown for four individuals with a deletion of *APOE* p.(Leu167del) (ε3) before and after splenectomy procedures. Data derived from meta-analysis (cases B, C, E and G, Table 1). Median and range depicted.

A total of six patients presented *APOE* variants in combination with splenomegaly: two patients had homozygous *APOE-ε1* variants, and all seven patients who were previously reported on in literature showed the same deletion of leucine 167 (NM_000041) on *APOE-ε3* (Table 1). Four out of seven of these patients (individuals A–C and F) also carried an APOE-ε2 isoform, which, in the adult males, generally led to higher levels in triglycerides and/or total cholesterol, even after the initiation of cholesterol-reducing treatment (with the exception of patient B). Seven out of nine patients suffered from either overweight or obesity (BMI ≥ 25 kg/m^2^) (both of our patients and individuals A–C, E and G). Information on BMI was not reported for the remaining two patients. Eight out of nine individuals (our two patients and individuals A, C–G) with splenomegaly showed dysbetalipoproteinemia (defined according to Berberich et al. [24] as LDL-C > 3.4 mmol/L and/or TG > 2.0 mmol/L and/or HDL-C < 0.9); these were mainly adult males. Five out of nine patients (individuals A and D–G), suffered (severe) ischemic heart disease from a relatively young age. Four out of the nine patients (individuals B, C, E and G) underwent splenectomy, and all of these individuals showed infiltration of sea blue histiocytes (foam cells) in spleen tissue. One of these patients also showed infiltration of foam cells in bone marrow. After splenectomy, median triglyceride values rose from 3.2 mmol/L to 16.4 mmol/L. The lipid profiles of individuals B, C, E and G, before and after splenectomy, are summarized in Figure 5. 

Eleven patients carried one or two *APOE-ε1* alleles (our two patients and individuals H-P; details in Table 2); in none of the patients reported in literature, spleen sizes were assessed. Five out of eleven individuals were homozygous for the *APOE-ε1* allele (our two patients and individuals I, J and P). The following characteristics seem to be associated with aggravated dyslipidemia: adulthood, male gender and homozygosity (as opposed to heterozygosity) for the *APOE-ε1* allele. Obesity also seems a predisposing factor for developing more severe dyslipidemia (with or without splenomegaly). This is in line with a higher risk of dyslipidemia in *APOE-ε2* homozygous individuals in the presence of obesity [25,26].

## 4. Discussion

In this article, we discuss a patient presenting with splenomegaly and several features indicative of a lysosomal storage disorder. The patient was eventually diagnosed with a macrophage storage disorder resulting from lipid accumulation due to a combination of a relatively common homozygous APOE isoform (APOE-ε2), combined with homozygosity for a rare amino acid change p.(Gly171Asp). This alteration of the APOE-ε2 isoform results in an isoform known as APOE-ε1, also referred to as the *Weisgraber allele*. Thus, the reported index patient is homozygous for *APOE-ε1*. The clinical consequences of heterozygosity for *APOE-ε1* have been reported in the literature (Table 2), but only three individuals homozygous for *APOE-ε1* were identified by our literature search, with limited details on the phenotypes of these individuals being found (Table 2, cases I, J and P). We suggest naming the lipid storage disorder caused by disruptive *APOE* variants ‘*inherited dyslipidemic splenomegaly’*, a slight variation on the name of the lipidemic splenomegaly used by Okorodudu et al. in 2013 [1]. 

To confirm the causal relationship between these changes in *APOE* and the established macrophage storage phenotype (enlarged spleen, foamy macrophages in bone marrow, elevated chitotriosidase activity), as well as to decipher the pathomechanism, we conducted several functional studies in monocytes. These showed lipid accumulation in monocytes of the *APOE-ε1* homozygous index patient. The patient’s monocytes showed an increased expression of CD11b, CCR2 and CD36, predominantly on classical monocytes. This increased expression could functionally lead to increased monocyte adhesion and migration into the intima. As a result of progressive lipid accumulation and activation, these macrophages convert into foam cells and can accumulate in tissues such as the spleen [27,28,29]. Of specific interest is the upregulation of CD36 on the surface of the patient’s *classical* and *mon 2/intermediate* monocyte fraction, given the role of CD36 in the uptake of oxidized LDL particles and entrapment of macrophages within atherosclerotic plaques, resulting in foam cell formation [30]. In addition, the expression of the anti-inflammatory marker CD163 in the patient’s *classical* and *mon 3/non-classical* monocyte subfractions was reduced. CD163 deficiency has been directly correlated to increased foam cell formation and plaque progression [31]. Moreover, expression of CD163 negatively regulates the release of interleukin-10 (IL-10), which in mouse models has been associated with reduced atherogenesis and an improvement of the stability of atherosclerotic plaques [32]. Conversely, the expression of TLR2 and TLR4 on monocytes (both increased during the development of atherosclerosis) was reduced in the patient’s *non-classical* monocyte fractions; however, this has been reported before in patients with chronic inflammation [33]. 

The findings from our literature study further support a causal connection between pathogenic homozygous and heterozygous rare variants in *APOE,* with a significant negative impact on APOE function and the presenting phenotype (dysbetalipoproteinemia and splenomegaly) (Table 1). In addition, this macrophage activation profile is consistent with an increased risk of early atherosclerosis, which is compatible with the finding of early-onset ischemic heart disease in the patients with p.(Leu167del) (*APOE-ε3*) reported in literature. Both the findings in our two patients and their family, as well as those reported in literature, suggest that obesity is an important modifier of the severity of the phenotype in individuals who are either heterozygous or homozygous for the *APOE-ε1* variant [25,26]. Obesity is known to alter lipid homeostasis, leading to an increased amount of triglyceride-rich lipoprotein particles in plasma, which are known to be cleared less efficiently in patients with disruptive *APOE* variants [34,35]. The additional predisposing factors for severe dyslipidemia in these patients appear to be the type of genetic variant (i.e., heterozygous vs. homozygous variants and location of the variant/residual APOE function), male gender and a post-splenectomy status [22,36,37]. The impact of these factors, as well as the influence of other genetic modifiers, will need to be confirmed in future studies with larger patient cohorts.

Diagnosing inherited dyslipidemic splenomegaly is important since it has consequences for the medical management of the index patient, namely treatment of the dyslipidemia, preventing/treating obesity and preventing splenectomy. Establishing a diagnosis also prevents further invasive diagnostics into potential hematological malignancies. Data from literature suggest that weight loss may also lead to reduction in spleen size (individual A, Table 1). Genetic counseling and the identification of other family members at risk is essential, as proper measures can prevent the early onset of atherosclerosis and cardiovascular events related to the dysbetalipoproteinemia caused by both the heterozygosity and homozygosity of *APOE-ε1,* as demonstrated by the index patient’s family history.

It is important to note that splenectomy is contra-indicated in this patient group. In all reported cases with *APOE* variants in which splenectomy was performed, the dyslipidemia was aggravated after the procedure (Figure 5 and Ai et al. [38]). This is in line with findings from other storage disorders, e.g., Gaucher disease and Niemann–Pick type B disease, where the manifestations in other organs (e.g., bone marrow or lungs) worsen after removal of the spleen, suggesting that the spleen functions as a sponge for the storage of excessive macrophages and foam cells [39,40,41]. The combined information from the index patient’s plasma profile (Appendix B Figure A1) and functional tests support the idea that there is a defect in both the clearance of VLDL from plasma whilst, at the same time, macrophages are more prone to ingest the defective APOE particles, together aggravating the dyslipidemic phenotype (Figure 1).

Although malfunctioning APOE isoforms are currently primarily perceived as risk factors for cardiovascular disease, *APOE* knock-out mice have confirmed a direct correlation between the loss of APOE and the development of splenomegaly [42]. Furthermore, it has been shown that some (both heterozygous and homozygous) *APOE* variant combinations are disruptive enough to lead to the development of a lipid storage phenotype in humans, characterized by dysbetalipoproteinemia, and splenomegaly, caused by accumulation of foam cells/sea-blue histiocytes in the spleen [1,8,12,21,43]. In the current report, we show that homozygosity of *APOE-ε1* can present with splenomegaly, where the human phenotype shows considerable overlap with lysosomal storage disorders, including the elevation of biomarkers (e.g., chitotriosidase activity, elevated in Gaucher and Niemann–Pick disease type B, and oxysterols, elevated in Niemann–Pick disease type B and C). The marked elevation of plasma PPCS in particular is important, as this was previously thought to be a Niemann–Pick type B and C specific marker but has now been shown to be elevated in several disorders that lead to the storage of lipids in macrophages [4,5,44,45]. 

## 5. Conclusions

In summary, patients that are under evaluation for a lysosomal storage disorder may actually suffer from dyslipidemic splenomegaly due to disruptive, heterozygous or homozygous *APOE* variants. This condition should therefore be added to the differential diagnosis, with a plasma lipid profile consistent with a dysbetalipoproteinemia serving as a diagnostic biomarker.

## Data Availability

The original contributions presented in the study are included in the article, further inquiries can be directed to the corresponding author.

## Abbreviations

The following abbreviations are used in this manuscript:

PPCS	N-palmitoyl-O-phosphocholine-serine
APOE	Apolipoprotein E
IMD	Inherited metabolic disorder
LSD	lysosomal storage disease
NPB/NPC	Niemann–Pick type B/Niemann–Pick type C
FACS	Flow cytometry
HDL	High-density lipoprotein
LDL	Low-density lipoprotein
VLDL	Very-low-density lipoprotein
TG	Triglycerides
TC	Total cholesterol
CAD	Coronary artery disease
LDLR	Low-density lipoprotein receptor
CD	Cluster of differentiation
CCR	Chemokine receptor
TLR	Toll-like receptor
Mon	Monocytes

## Appendix A

**Table A1:** Lysosomal storage disorders associated with unexplained splenomegaly in adults.

Defect	Disorder	Symptoms in adult Phenotype	Dyslipidemia	Main Biomarker	Gene	Prevalence *
Glucocerebrosidase deficiency/Saposin C deficiency	**Gaucher disease**	Hepatosplenomegaly, skeletal symptoms, cytopenia, neurological symptoms (type II/III only)	Y (subtle)	Chitotriosidase, glucosylsphingosine	*GBA* or *PSAP*	1–9/100,000
Acid sphingomyelinase deficiency	**Niemann–Pick type B**	Hepatosplenomegaly, interstitial lung disease	Y	Lyso-sphingomyelin (lyso-SM), *N-palmitoyl-O-phosphocholineserine (PPCS*)	*SMPD1*	1–9/100,000
Cholesterol trafficking disorder	**Niemann–Pick type C**	Hepatosplenomegaly, ataxia, hypotonia, vertical supranuclear gaze palsy, dysphagia, dysarthria, respiratory infections/failure, liver failure	Y	*N-palmitoyl-O-phosphocholineserine (PPCS*), Cholestane-3β,5α,6β-triol	*NPC1* or *NPC2*	1–9/100,000
Lysosomal β-mannosidase deficiency	**β-Mannosidosis**	Mild cognitive impairement, psychiatric symptoms, hearing loss, recurrent respiratory infections, coarse facial features, skeletal abnormalities hepatosplenomegaly	N	Oligosaccharides	*MANBA*	1/1,000,000
α-neuraminidase deficiency	**Sialidosis**	Seizures, hyperreflexia, ataxia, myoclonus, cherry-red maculas, hepatosplenomegaly, dysostosis multiplex	N	Oligosaccharides	*NEU1*	<1/1,000,000
Lysosomal acid lipase (LAL) deficiency	**Cholesterol ester storage disease (CESD)**	Hepatosplenomegaly, dyslipidemia, liver dysfunction, premature atherosclerosis	Y (marked)	Transaminases, total cholesterol, LDL-cholesterol, HDL-cholesterol and triglycerides	*LIPA*	1/40,000–300,000
High-density lipoprotein (HDL) deficiency	**Tangier disease**	Enlarged orange/yellow tonsils, premature atherosclerosis, hepatosplenomegaly, neuropathy, cytopenia, corneal clouding, dyslipidemia	Y (marked)	HDL-cholesterol/apolipoprotein A-I (ApoA1)	*ABCA1*	<1/1,000,000
LPL deficiency	**Chylomicronemia syndrome**	Acute pancreatitis, hepatosplenomegaly, xanthoma	Y (marked)	Triglycerides	*LPL*, *APOC2*, *GPIHBP1*, *LMF1*, *APOA5*	1–9/1,000,000
Partial LCAT deficiency (**Fish-eye disease**)	**Lecithin-cholesterol acyltransferase (LCAT)**	Corneal opacities, progressive renal disease, hemolytic anemia, hepatosplenomegaly	Y (marked)	HDL-cholesterol/apolipoprotein A-I (ApoA1)/primary HDL proteins	*LCAT*	<1/1,000,000

* source www.orpha.net, accessed on the 3 June 2024.

## Appendix B

**Table A2:** Antigen overview.

Target Antigen	Vendor or Source	Catalog #	Working Concentration	Lot #
**CD91-FITC**	BD Biosciences, Franklin Lakes, NJ, USA	550,496	1:500	5,069,533
**CCR2-Alexa fluor 647**	BD Biosciences	558,406	1:50	6,105,900
**CD11c-APC**	BD Biosciences	559,877	1:50	6,187,944
**CD36-APC**	BD Biosciences	550,956	1:100	5,240,938
**CD18-APC**	BD Biosciences	551,060	1:25	4,324,584
**TLR2-FITC**	BioLegend, San Diego, CA, USA	121,806	1: 100	B218,515
**TLR4-PE**	BioLegend	312,806	1:10	B252,428
**CD11b-PE**	BD Biosciences	555,388	1:25	9,045,644
**CD163**	BD Biosciences	556,018	1:25	36,295
**HLA-DR-PercpC5.5**	BD Biosciences	560,652	1: 50	9,074,606
**CD14-PEcy7**	BD Biosciences	557,742	1: 50	8,019,559
**CD16-APC-Cy7**	BD Biosciences	560,195	1: 50	9,037,577
**Nanogram IVIG**	Sanquin, Amsterdam, The Netherlands	8,717,185,830,262	1:50	18625H462B

**Table A3:** Full overview of detected (potentially pathogenic) variants.

Gene name	phyloP	CADD	SpliceAI-G	OMIM_DISEASE	ClinVar	ClinVar Accession	Zygosity	gnomAD-G AF (%)	Hgvsg	Hgvsc	Hgvsp	Mutation Taster	PolyPhen2	SIFT
***APOE***	2.669	24.3	0	Hyperlipoproteinemia, type III, 617347 (3); {Coronary artery disease, severe, susceptibility to}, 617347 (3); {?Alzheimer disease, protection against, due to APOE3-Christchurch}, 607822 (3), Autosomal dominant; Lipoprotein glomerulopathy, 611771 (3); Sea-blue histiocyte disease, 269600 (3), Autosomal recessive; {?Macular degeneration, age-related}, 603075 (3), Autosomal dominant; Alzheimer disease 2, 104310 (3), Autosomal dominant	VUS	VCV000478904.7	Homozygous	0.0128	19:g.45411987G>A	NM_001302688.2(APOE):c.512G>A	p.(Gly171Asp)			Tolerated
***APOE***	0.344	25	0	Hyperlipoproteinemia, type III, 617347 (3); {Coronary artery disease, severe, susceptibility to}, 617347 (3); {?Alzheimer disease, protection against, due to APOE3-Christchurch}, 607822 (3), Autosomal dominant; Lipoprotein glomerulopathy, 611771 (3); Sea-blue histiocyte disease, 269600 (3), Autosomal recessive; {?Macular degeneration, age-related}, 603075 (3), Autosomal dominant; Alzheimer disease 2, 104310 (3), Autosomal dominant	Inconsistent	VCV000017848.16	Homozygous	8.27	19:g.45412079C>T	NM_001302688.2(APOE):c.604C>T	p.(Arg202Cys)			Deleterious
***BAG3***	3.719	42	0	Cardiomyopathy, dilated, 1HH, 613881 (3), Autosomal dominant; Myopathy, myofibrillar, 6, 612954 (3), Autosomal dominant			Homozygous	ND	10:g.121436630C>T	NM_004281.4(BAG3):c.1564C>T	p.(Gln522*)			
***MYH3***	0.196	11.71	0	Contractures, pterygia, and spondylocarpotarsal fusion syndrome 1B, 618469 (3), Autosomal recessive; Arthrogryposis, distal, type 2B3 (Sheldon-Hall), 618436 (3), Autosomal dominant; Arthrogryposis, distal, type 2A (Freeman-Sheldon), 193700 (3), Autosomal dominant; Contractures, pterygia, and spondylocarpostarsal fusion syndrome 1A, 178110 (3), Autosomal dominant			Heterozygous	ND	17:g.10552930_10552931ins121	NM_002470.4(MYH3):c.605_606ins121	p.(Gly203Profs*78)			
***NPC1***	0.437	8.503	0	Niemann-Pick disease, type D, 257220 (3), Autosomal recessive; Niemann-Pick disease, type C1, 257220 (3), Autosomal recessive	Benign	VCV000378276.8	Heterozygous	0.21	18:g.21148768T>C	NM_000271.5(NPC1):c.463+19A>G	p.?			
***SACS***	6.4	21.6	0	Spastic ataxia, Charlevoix-Saguenay type, 270550 (3), Autosomal recessive	Likely benign/VUS	VCV000212109.41	Homozygous	0.175	13:g.23907033G>A	NM_014363.6(SACS):c.10982C>T	p.(Ala3661Val)	Benign	Probably damaging	Deleterious
***SIPA1L3***	1.963	20.2	0	?Cataract 45, 616851 (3), Autosomal recessive			Homozygous	0.00319	19:g.38573424T>C	NM_015073.3(SIPA1L3):c.1219T>C	p.(Cys407Arg)	Benign	Benign	Tolerated
***VHL***	0.256	20.9	0	Pheochromocytoma, 171300 (3), Autosomal dominant; Erythrocytosis, familial, 2, 263400 (3), Autosomal recessive; von Hippel-Lindau syndrome, 193300 (3), Autosomal dominant; Renal cell carcinoma, somatic, 144700 (3); Hemangioblastoma, cerebellar, somatic (3)	VUS	VCV000132707.13		ND	3:g.10183635C>A	NM_000551.4(VHL):c.104C>A	p.(Ala35Asp)	Benign	Possibly damaging	Deleterious
***VHL***	0.125	21.6	0	Pheochromocytoma, 171300 (3), Autosomal dominant; Erythrocytosis, familial, 2, 263400 (3), Autosomal recessive; von Hippel-Lindau syndrome, 193300 (3), Autosomal dominant; Renal cell carcinoma, somatic, 144700 (3); Hemangioblastoma, cerebellar, somatic (3)	VUS	VCV000216476.14		ND	3:g.10183545C>T	NM_000551.4(VHL):c.14C>T	p.(Ala5Val)	Benign	Possibly damaging	Tolerated
***BEX5***	2.518	22.4	0				Hemizygous	ND	X:g.101409056A>C	NM_001159560.2(BEX5):c.182T>G	p.(Ile61Arg)	Benign	Probably damaging	Deleterious
***CAPN12***	1.574	24.2	0				Homozygous	0.00637	19:g.39221807G>C	NM_144691.4(CAPN12):c.2014C>G	p.(Arg672Gly)	Benign	Probably damaging	Deleterious
***DPY19L3***	4.949	22.5	0				Homozygous	0.00956	19:g.32902201G>T	NM_207325.3(DPY19L3):c.160G>T	p.(Ala54Ser)	Benign	Benign	Tolerated
***TAF5L***	6.292	24.7	0				Homozygous	0.0987	1:g.229730691T>C	NM_014409.4(TAF5L):c.1123A>G	p.(Thr375Ala)	Benign	Probably damaging	Tolerated
***TNK2***	5.948	25.9	0				Heterozygous	0.0128	3:g.195594795G>A	NM_001387707.1(TNK2):c.2470C>T	p.(Arg824Trp)			Deleterious
***TNK2***	3.788	25.9	0				Heterozygous	0.0321	3:g.195594882G>A	NM_001387707.1(TNK2):c.2383C>T	p.(Arg795Trp)			Deleterious
***ZNF532***	0.068	11.38	0				Heterozygous	0.271	18:g.56586091C>T	NM_018181.6(ZNF532):c.572C>T	p.(Thr191Met)	Benign	Benign	Tolerated
***ZNF532***	6.492	24.3	0				Heterozygous	0.0159	18:g.56651600G>A	NM_018181.6(ZNF532):c.3808G>A	p.(Ala1270Thr)	Benign	Possibly damaging	Tolerated

ND = not detected.

## Appendix C

Raw facs analysis data of the expression of monocyte surface markers and Intracellular lipid droplet accumulation.

**Table A4:** FACS data of expression of monocyte surface marker CD11b.

Total		Mon 1		Mon 2		Mon 3		
Ctrl	Proband	Ctrl	Proband	Ctrl	Proband	Ctrl	Proband	
**3216.7**	4718.4	3620	5059.1	4345	5262	642	595.9	
**3247**		4086		4006		554		
**2700.9**		2745.6		3845		960.1		
**3216.7**		3620		4006		642		**Median**

**Table A5:** FACS data of expression of monocyte surface marker CCR2.

Total		Mon 1		Mon 2		Mon 3		
Ctrl	Proband	Ctrl	Proband	Ctrl	Proband	Ctrl	Proband	
**481**	677	527	703	283.4	588	38	29	
**477**		540		274		117		
**494.1**		510.5		204		30		
**481**		527		274		38		**Median**

**Table A6:** FACS data of expression of monocyte surface marker CD163.

Total		Mon 1		Mon 2		Mon 3		
Ctrl	Proband	Ctrl	Proband	Ctrl	Proband	Ctrl	Proband	
**165.7**	121.4	178	125.1	143	164	−2	9.2	
**208**		223		212		92.9		
**200.9**		206.6		141		23		
**200.9**		206.6		143		23		**Median**

**Table A7:** FACS data of expression of monocyte surface marker CD18.

Total		Mon 1		Mon 2		Mon 3		
Ctrl	Proband	Ctrl	Proband	Ctrl	Proband	Ctrl	Proband	
**1271**	1582	1210	1586	2094.4	2296	2081	1435	
**1628**		1550		2730		1993		
**817.1**		800.5		1311		1190		
**1271**		1210		2094.4		1993		**Median**

**Table A8:** FACS data of expression of monocyte surface marker CD11c.

Total		Mon 1		Mon 2		Mon 3		
Ctrl	Proband	Ctrl	Proband	Ctrl	Proband	Ctrl	Proband	
**385**	461	336	465	931.4	1087	1094	262	
**428**		379		1050		706		
**248.1**		237.5		601		523		
**385**		336		931.4		706		**Median**

**Table A9:** FACS data of expression of monocyte surface marker TLR2.

Total		Mon 1		Mon 2		Mon 3		
Ctrl	Proband	Ctrl	Proband	Ctrl	Proband	Ctrl	Proband	
**2658**	1996	2698	2093	3620	2462	1954	241	
**1763**		1855		2457		1140		
**1735**		1742		2361		1152		
**1763**		1855		2457		1152		**Median**

**Table A10:** FACS data of expression of monocyte surface marker TLR4.

Total		Mon 1		Mon 2		Mon 3		
Ctrl	Proband	Ctrl	Proband	Ctrl	Proband	Ctrl	Proband	
**3128.7**	2521.4	3161	2573.1	3779	3823	2519	1212.9	
**3387**		3541		3994		2427		
**1416.9**		1407.6		1896		1399.1		
**3128.7**		3161		3779		2427		**Median**

**Table A11:** FACS data of expression of monocyte surface marker CD36.

Total		Mon 1		Mon 2		Mon 3		
Ctrl	Proband	Ctrl	Proband	Ctrl	Proband	Ctrl	Proband	
**3122**	3835	3610	4151	3644.4	4594	296	72	
**1716**		2118		1636		287		
**1593.1**		1654.5		1612		194		
**1716**		2118		1636		287		**Median**

**Table A12:** FACS data of intracellular lipid droplet accumulation.

Ctrl	Proband	
**2.87**	4.47	
**2.29**		
**4.41**		
**2.87**		**Median**
**3.19**		**Mean**
